# Early recognition of peripheral intravenous catheter failure using serial ultrasonographic assessments

**DOI:** 10.1371/journal.pone.0253243

**Published:** 2021-06-16

**Authors:** Amit Bahl, Steven Johnson, Nicholas Mielke, Patrick Karabon

**Affiliations:** 1 Department of Emergency Medicine, Beaumont Hospital, Royal Oak, Michigan, United States of America; 2 Department of Emergency Medicine, Oakland University William Beaumont School of Medicine, Rochester, Michigan, United States of America; 3 Department of Statistics, Oakland University William Beaumont School of Medicine, Rochester, Michigan, United States of America; Ohio State University Wexner Medical Center Department of Surgery, UNITED STATES

## Abstract

**Objective:**

Peripheral intravenous catheter (PIVC) failure occurs frequently, but the underlying mechanisms of failure are poorly understood. We aim to identify ultrasonographic factors that predict impending PIVC failure prior to clinical exam.

**Methods:**

We conducted a single site prospective observational investigation at an academic tertiary care center. Adult emergency department (ED) patients who underwent traditional PIVC placement in the ED and required admission with an anticipated hospital length of stay greater than 48 hours were included. Ongoing daily PIVC assessments included clinical and ultrasonographic evaluations. The primary objective was to identify ultrasonographic PIVC site findings associated with an increased risk of PIVC failure. The secondary outcome was to determine if ultrasonographic indicators of PIVC failure occurred earlier than clinical recognition of PIVC failure.

**Results:**

In July and August of 2020, 62 PIVCs were enrolled. PIVC failure occurred in 24 (38.71%) participants. Multivariate logistic regression demonstrated that the presence of ultrasonographic subcutaneous edema [AOR 7.37 (1.91, 27.6) p = 0.0030] was associated with an increased likelihood of premature PIVC failure. Overall, 6 (9.67%) patients had subcutaneous edema present on clinical exam, while 35 (56.45%) had subcutaneous edema identified on ultrasound. Among patients with PIVC failure, average time to edema detectable on ultrasound was 46 hours and average time to clinical recognition of failure was 67 hours (P = < 0.0001).

**Conclusions:**

Presence of subcutaneous edema on ultrasound is a strong predictor of PIVC failure. Subclinical subcutaneous edema occurs early and often in the course of the PIVC lifecycle with a predictive impact on PIVC failure that is inadequately captured on clinical examination of the PIVC site. The early timing of this ultrasonographic finding provides the clinician with key information to better anticipate the patient’s vascular access needs. Further research investigating interventions to enhance PIVC survival once sonographic subcutaneous edema is present is needed.

## Introduction

The placement of peripheral intravenous catheters (PIVC) is the most commonly performed invasive procedure in the acute care clinical setting with over 300 million PIVCs inserted annually in the United States alone [1–3]. Up to 90% of hospitalized patients require a PIVC for therapy. Unfortunately, PIVCs have high failure rates ranging from 36% to 63%. This high rate of failure leads to significant patient safety and cost implications [2–4]. Patients may suffer a multitude of sequelae from PIVC failure including: extravasation with skin necrosis, catheter-associated bloodstream infections, interruption of medical therapies, venous depletion, and longer hospital stays [5–7].

A better understanding of the etiology of PIVC failure is critical to improving patient outcomes. Most of the existing literature highlights the various causes of PIVC failure, such as phlebitis, infiltration, or occlusion. Our current understanding of PIVC failure relies on mostly external observation of the PIVC site for these various complications. As the presence of these complications generally equates to the need for catheter removal, once the complication is externally evident, little can be done to reverse course [8]. This current assessment method does not capture the evolution and progression of the cause that ultimately leads to catheter failure until it is too late. Some recent exploratory research using ultrasound technology determined that changes in the vein and soft tissue often occur after PIVC insertion [9]. Internally, venous and soft tissue changes may include: narrowing of the vein wall, vein wall thickening, subcutaneous edema, and presence of thrombus. These changes can occur even in the absence of any clinical signs or symptoms evident on external exam [9]. While the literature has demonstrated that ultrasound is capable of identifying these subcutaneous changes, how well they correlate with impending or ongoing PIVC failure remains unclear. Further, the timing of the sonographic features of impending PIVC failure remains unknown. It is possible that earlier identification of impending failure may allow for meaningful intervention to reverse course or salvage a PIVC and quantification of this time interval is a necessary first step. At a minimum, these findings may help the clinician anticipate future vascular access needs.

The primary goal of this investigation is to determine sonographic predictors of PIVC failure. Further, we aim to determine if the appearance of sonographic features of failure precedes clinical recognition of catheter failure.

## Materials and methods

### Study design, setting, and selection of participants

This study was a prospective observational investigation of PIVC failure. The study was conducted at a large 1100 bed tertiary care center with an annual ED census of greater than 130,000 visits. The Beaumont Health Institutional Review Board (IRB) approved this study.

Study investigators recruited a convenience sample of ED patients meeting inclusion criteria. Patients aged at least 18 years with anticipated hospitalization of greater than 48 hours as well as a PIVC placed using direct visualization and/or palpation were eligible participants. Patients admitted to the high acuity progressive and intensive care units were specifically targeted to increase the likelihood of meeting the minimum hospital length of stay goal of 48 hours. Patients were excluded if they voluntarily withdrew or were cognitively impaired. If the PIVC was inserted with ultrasound guidance or if the first sonographic assessment could not be conducted within 24 hours of PIVC placement, then the patient was not eligible for enrollment. Verbal informed consent was obtained for all subjects prior to enrollment in the study.

### Study procedure

After patient enrollment, researchers performed an initial assessment of the PIVC site and abstracted data from the patient’s electronic medical record (EMR). The following pertinent demographic and clinical data were abstracted from the EMR: age, body mass index, admission blood pressure, admission heart rate, gender, smoking history, pre-existing medical conditions (diabetes, deep vein thrombosis history, clotting disorder, cancer), and use of anticoagulant medications.

PIVC function was confirmed by clinical assessment (per institutional standard), in which a functional PIVC can be flushed without resistance and shows no external signs of unresolvable complication. PIVC complications include: pain, tenderness, redness, and leaking or swelling around the PIVC site. The investigator performed a sonographic evaluation of the PIVC and surrounding area using a uniform scanning technique that has been previously described in the literature [10]. Study investigators trained in using ultrasound were responsible for obtaining images. The Mindray M7 Ultrasound Machine with a 14 MHz high-frequency linear array transducer was used for all sonographic evaluations. After a small amount of sterile gel was placed on the non-bordered transparent dressing proximal to the PIVC insertion site, the PIVC and surrounding tissue was scanned proximally (towards the heart) 10 cm (length) x 5 cm (width) in short axis extending from the hub of the PIVC. Similar scanning was performed over the same area in the long axis. S1 Appendix demonstrates the scan area. Adequate placement of the PIVC within the vein was confirmed using ultrasound. Sterile ultrasound gel was cleaned off the PIVC site and skin after the imaging took place.

A series of video clips (five seconds duration) and still images of the scan area were recorded. All ultrasound data was saved and archived in QPath, a secure and Health Insurance Portability and Accountability Act (HIPPA) compliant storage warehouse for review and interpretation by the Emergency Ultrasound Director. The following measurements were made by post-processing of the original images: catheter-to-vein ratio, length of catheter in vein, angle of insertion, angle of distal tip against vessel wall, vein wall thickness, distance of catheter tip to vessel wall, degree of catheter kinking, and size of thrombus formation (S2 Appendix).

Investigators performed follow-up ultrasound and clinical assessments on all catheters daily for the life of the PIVC. At each follow-up interval, the researcher documented the time of evaluation and performed a sonographic assessment using the identical method as described above. Subsequent images and videos were also saved, archived, and reviewed as described above. Follow-up ultrasound data included the additional variables: vein wall thickness, distance of catheter tip to vessel wall, degree of catheter kinking, as well as the assessment of thrombus and subcutaneous edema (S3 Appendix). Subcutaneous edema was defined as presence of fluid within the subcutaneous tissue adjacent to the vein of interest. Sonographically, this appears as a cobblestone pattern.

Clinical staff document the functional status of PIVCs in the EMR as a standard of care measure within our institution. Daily assessment of catheter function was accomplished by reviewing this documentation in the EMR for any notation of catheter failure or complications. If the investigators had any questions or concerns regarding the functionality of the PIVC, clinical staff was brought to the bedside to reassess functionality of the PIVC. If the catheter failed or was removed prior to a follow-up assessment, the PIVC failure time, assessment of failure, and reason for line removal was obtained through EMR review and discussion with the nursing staff when possible.

All medications administered through each catheter were queried and cross-referenced against known irritants and vesicants, as defined by the Infusion Nursing Society [11]. Frequency of administration and dosages were recorded. Beyond vesicants and irritants, the number of overall catheter events was also recorded. A catheter event was defined as any instance where fluid was administered through the catheter regardless of quantity or composition. However, flushing was considered a component of routine care and PIVC maintenance and was not considered an independent event.

### Outcome measures

The primary objective was to identify ultrasonographic PIVC site findings associated with an increased risk of PIVC failure. The secondary outcome was to determine if ultrasonographic indicators of PIVC failure occurred earlier than clinical recognition of PIVC failure. PIVC failure was defined as the presence of any irreversible PIVC-related complication on a traditional clinical external exam.

### Statistical rationale and analysis

No formal sample size calculation was conducted for this investigation. Given the paucity of existing evidence on this topic, we had difficulty substantiating any assumptions and making a precise calculation. Instead, enrollment was based upon feasibility during the study period.

Continuously measured variables were displayed in terms of mean/average with standard deviation while categorical variables were displayed as frequencies with percentages in parentheses. Univariate, or unadjusted, analysis was performed. Continuous variables were stratified by PIVC failure/survival and compared using a Two Samples Independent T-Tests. Categorical variables also were stratified by PIVC failure/survival and compared using Chi-Square tests. Odds Ratios (OR) with corresponding 95% Confidence Intervals (95% CI) also were displayed for categorical variables. In addition, univariate logistic regression models were used and results were displayed in terms of Odds Ratios (OR) with corresponding 95% CI and P-Values. Kaplan-Meier Curves were graphically generated to show the difference in time-to-event outcomes on selected characteristics. Pearson’s correlation and a Paired T-Test were used to assess the association between Time to Subcutaneous Edema and Time to Failure.

Multivariate/adjusted models also were generated as part of this study. Variables included in these models were chosen by all authors based on clinical rationale and the univariate/unadjusted findings. Firth’s Penalized Likelihood was employed to mitigate the potential bias caused by the relatively small sample [12]. A multivariate logistic regression model was used. Effect sizes were shown in terms of Adjusted Odds Ratios (AOR) with 95% CI and P-Values for the logistic model.

P-Value < 0.05 indicates a statistically significant finding. All significant findings represent associations as no formal attempts were made to identify cause-and-effect, or causal, relationships. Data was entered and managed in RedCap and all analysis was performed in SAS 9.4 (SAS Institute Inc., Cary, NC, USA).

## Results

In July and August of 2020, 77 patients consented for the study; of these, 15 participants were excluded. 12 of these patients were lost to follow up (5 PIVCs failed, and 7 patients were discharged prior to the first ultrasound evaluation). Additionally, 2 PIVCs were excluded due to incomplete clinical PIVC documentation and one patient voluntarily withdrew from the study. Of the remaining 62 PIVCs, 24 (38.7%) met the criteria for premature failure and 38 (61.2%) survived to completion of therapy. The mean catheter dwell time was 76.42 hours (SD = 66.60).

Patient demographics and comorbidities were similar between the catheters that failed and survived to completion of therapy (all P ≥ 0.05) (Table 1). IV Vesicant/Irritant administration was more common in catheters who failed (P = 0.0064) and the average number of catheter events in the survival group was 3.39, which was significantly less than the failure group at 5.58 (P = 0.0011). The average percent of days idle for the survival group was 38%, compared to 6% in the failure group (P < 0.0001) (Table 2).

**Table 1:** Patient characteristics, comorbidities, vital signs, lab values, & IV insertion characteristics.

		All PIVCs	Failed	Survived	P-Value
		(n = 62)	(n = 24)	(n = 38)	
**Patient Characteristics**					
Age of Patient (Years)					
	Mean (Standard Deviation)	67.18 (19.25)	65.79 (18.12)	68.05 (20.12)	0.6561
Gender					
	Male	32 (51.61%)	14 (43.75%)	18 (56.25%)	
	Female	30 (48.39%)	10 (33.33%)	20 (66.67%)	0.4178
Body Mass Index (BMI) of Patient					
	Mean (Standard Deviation)	28.22 (6.82)	29.22 (7.26)	27.58 (6.56)	0.3632
**Comorbidities**					
History of Smoking					
	No	32 (51.61%)	13 (40.63%)	19 (59.38%)	
	Yes	30 (48.39%)	11 (36.67%)	19 (63.33%)	0.7588
History of Diabetes					
	No	45 (72.58%)	15 (33.33%)	30 (66.67%)	
	Yes	17 (27.42%)	9 (52.94%)	8 (47.06%)	0.1735
History of Active Cancer					
	No	54 (87.10%)	22 (40.74%)	32 (59.26%)	
	Yes	8 (12.90%)	2 (25.00%)	6 (75.00%)	0.482
History of Previous DVT					
	No	55 (88.71%)	20 (36.36%)	35 (63.64%)	
	Yes	7 (11.29%)	4 (57.14%)	3 (42.86%)	0.3243
Personal History of Clotting Disorder					
	No	54 (87.10%)	19 (35.19%)	35 (64.81%)	
	Yes	8 (12.90%)	5 (62.50%)	3 (37.50%)	0.1774
Currently on Anticoagulant Medication					
	No	45 (72.58%)	18 (40.00%)	27 (60.00%)	
	Yes	17 (27.42%)	6 (35.29%)	11 (64.71%)	0.7676
**Vital Signs at Time of Admission**					
Systolic Blood Pressure at Admission					
	Mean (Standard Deviation)	131.95 (20.82)	126.08 (18.68)	135.66 (21.47)	0.0775
Heart Rate at Admission					
	Mean (Standard Deviation)	84.56 (22.14)	98.75 (22.01)	75.61 (17.15)	< 0.0001
**IV Insertion Characteristics**					
Laterality of Successful Cannulation					
	Left	27 (43.55%)	12 (44.44%)	15 (55.56%)	0.4282
	Right	35 (56.45%)	12 (34.29%)	23 (65.71%)	
Location of IV					
	Antecubital	47 (75.81%)	20 (42.55%)	27 (57.45%)	
	Forearm	15 (24.19%)	4 (26.67%)	11 (73.33%)	0.318
Catheter-to-vein ratio					
	Mean (Standard Deviation)	0.36 (0.15)	0.37 (0.16)	0.35 (0.15)	0.5999
Length of Catheter in Vein (long axis) (cm)					
	Mean (Standard Deviation)	1.98 (0.37)	1.95 (0.44)	2.00 (0.32)	0.5949
Angle of Insertion (long axis) (degrees)					
	Mean (Standard Deviation)	15.38 (6.47)	15.00 (4.58)	15.61 (7.43)	0.6953
Angle of Distal Tip Against Vessel Wall (long axis) (degrees)					
	Mean (Standard Deviation)	6.10 (5.13)	5.67 (5.28)	6.37 (5.09)	0.6039

**Table 2:** Daily IV characteristics, clinical symptoms, sonographic findings, IV infusate administration, and IV usage characteristics.

		All Lines	Failed	Survived	P-Value
		(n = 62)	(n = 24)	(n = 38)	
**Daily IV Characteristics**					
Vein Wall Thickness (short axis) (cm)					
	Mean (Standard Deviation)	0.04 (0.01)	0.04 (0.02)	0.05 (0.01)	0.5931
Distance of Catheter Tip to Vessel Wall (cm)					
	Mean (Standard Deviation)	0.04 (0.04)	0.03 (0.03)	0.04 (0.05)	0.0561
Degree of Catheter Kinking (long axis) (degrees)					
	Mean (Standard Deviation)	4.17 (3.31)	4.36 (3.28)	4.06 (3.37)	0.7316
**Sonographic Findings**					
Presence of subcutaneous edema					
	No	27 (43.55%)	4 (14.81%)	23 (85.19%)	
	Yes	35 (56.45%)	20 (57.14%)	15 (42.86%)	0.002
Time to Edema		**(n = 35)**	**(n = 20)**	**(n = 15)**	
	Mean (Standard Deviation)	46.92 (52.32)	39.17 (34.61)	57.25 (69.44)	0.366
	< 24 Hours	8 (22.86%)	6 (75.00%)	2 (25.00%)	
	24–48 Hours	18 (51.43%)	10 (55.56%)	8 (44.44%)	0.5333
	> 48 Hours	9 (25.71%)	4 (44.44%)	5 (55.56%)	
Peri/Tip Thrombus		**(n = 54)**	**(n = 22)**	**(n = 32)**	
	Yes	40 (74.07%)	17 (42.50%)	23 (57.50%)	0.6941
	No	14 (25.93%)	5 (35.71%)	9 (64.29%)	
Time to Thrombus		**(n = 54)**	**(n = 22)**	**(n = 32)**	
	Mean (Standard Deviation)	26.22 (19.88)	23.57 (14.94)	28.05 (22.71)	0.3862
	< 24 Hours	28 (51.85%)	12 (42.86%)	16 (57.14%)	
	24 Hours +	26 (48.15%)	10 (38.46%)	16 (61.54%)	0.7535
**IV Infusate Administration**					
IV Medication Administration					
	No	11 (17.74%)	1 (9.09%)	10 (90.91%)	
	Yes	51 (82.26%)	23 (45.10%)	28 (54.90%)	0.0661
IV Vesicant/Irritant Administration					
	No	11 (17.74%)	9 (81.82%)	2 (18.18%)	0.0064
	Yes	51 (82.26%)	15 (29.41%)	36 (70.59%)	
**IV Usage Characteristics**					
Catheter Dwell Time (Hours)					
	Mean (Standard Deviation)	76.42 (66.60)	66.85 (44.34)	82.47 (77.42)	0.3171
Percent of Days Idle (%)					
	Mean (Standard Deviation)	26% (31%)	6% (17%)	38% (32%)	< 0.0001

**Fig 1:**
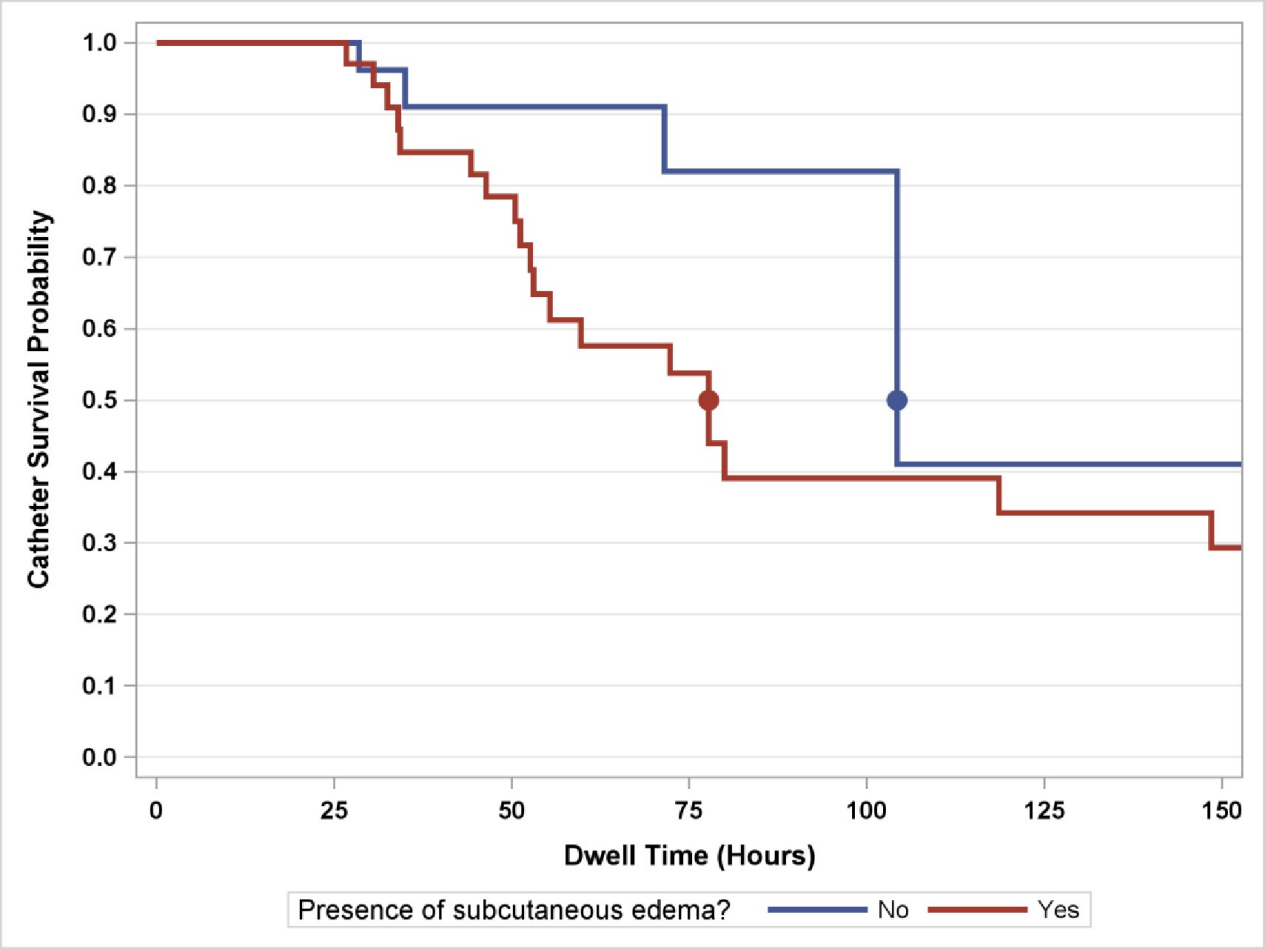
Kaplan-Meier survival curve estimates for PIVC survival. The median survival time was 77.69 hours for participants with subcutaneous edema and 104.23 hours for participants without subcutaneous edema.

Our analysis showed a significant association between ultrasonographic signs of subcutaneous edema and catheter failure. While 57.14% of PIVC’s that had subcutaneous edema identified by ultrasound failed, only 14.81% of PIVC’s without these findings failed (P = 0.0020) (Fig 1). Unadjusted for other factors, logistic regression analysis demonstrated that ultrasonographic subcutaneous edema was associated with 6.91-fold greater odds of catheter failure (P = 0.0020). Multivariate logistic regression analysis, which was adjusted for other ultrasonographic factors, demonstrated subcutaneous edema was independently associated with 7.37-fold greater odds of premature catheter failure (P = 0.0030). No other sonographic factors included in the multivariate analysis (Thrombosis, Catheter-to-Vein Ratio, Distance Catheter Tip to Vessel Wall, or Vein Wall Thickness) demonstrated significance (all P ≥ 0.05) (Table 3).

**Table 3:** Univariate and multivariate analysis.

		Univariate		Multivariate	
		OR (95% CI)	P-Value	AOR (95% CI)	P-Value
**Patient Characteristics**					
Gender					
	Male	Reference Group			
	Female	0.65 (0.23, 1.83)	0.4178		
**Comorbidities**					
History of Smoking					
	No	Reference Group			
	Yes	0.85 (0.31, 2.37)	0.7588		
History of Diabetes					
	No	Reference Group			
	Yes	2.20 (0.71, 6.85)	0.1735		
History of Active Cancer					
	No	Reference Group			
	Yes	0.56 (0.11, 2.86)	0.482		
History of Previous DVT					
	No	Reference Group			
	Yes	2.23 (0.45, 10.9)	0.3243		
Personal History of Clotting Disorder					
	No	Reference Group			
	Yes	2.86 (0.62, 13.2)	0.1774		
Currently on Anticoagulant Medication					
	No	Reference Group			
	Yes	0.84 (0.27, 2.67)	0.7676		
**IV Insertion Characteristics**					
Laterality of Successful Cannulation					
	Left	1.52 (0.54, 4.25)	0.4282		
	Right	Reference Group			
Location of IV					
	Antecubital	Reference Group			
	Forearm	0.53 (0.15, 1.86)	0.318		
Catheter-to-Vein Ratio					
	Ratio ≥ 33%	1.23 (0.44, 3.42)	0.6935	1.50 (0.44, 5.11)	0.5165
	Ratio < 33%	Reference Group		Reference Group	
**Sonographic Findings**					
Distance Catheter Tip to Vein Wall					
	0 only	Reference Group		Reference Group	
	0 and > 0	0.80 (0.25, 2.50)	0.6962	0.63 (0.17, 2.34)	0.4929
	> 0 only	0.48 (0.08, 2.89)	0.4187	0.47 (0.06, 3.89)	0.4869
Vein Wall Thickness					
	Thickness ≥ 0.04cm	0.62 (0.22, 1.77)	0.3721	0.39 (0.11, 1.42)	0.1528
	Thickness < 0.04cm	Reference Group		Reference Group	
Subcutaneous Edema					
	Yes	6.91 (2.03, 23.5)	0.002	7.37 (1.97, 27.6)	0.003
	No	Reference Group		Reference Group	
Presence of Thrombus					
	Yes	1.29 (0.37, 4.51)	0.6941	1.76 (0.24, 13.0)	0.5729
	No	Reference Group		Reference Group	

Among failed catheters, the etiology of failure was noted in the EMR for 20 (83.33%) of the cases. Overall, these etiologies included 3 (12.5%) dislodgement events, 6 (25%) cases of infiltration, 1 (4.16%) catheter kinking issue, 6 (25%) cases where the PIVC was leaking, 4 (16.66%) failed due to pain at the site, and in 4 (16.66%) cases the cause of failure was not documented.

While the average time to the clinical recognition of PIVC failure was 68.26 hours, the average time to ultrasonographic evidence of subcutaneous edema was only 39.17 hours in lines where we had information for time to failure and time to subcutaneous edema noted in the data (n = 20). On average, edema was noted 29.09 hours before failure (P = < 0.0001).

## Discussion

Existing prediction models for PIVC failure are sparse.^1^ To our knowledge, this is the first investigation that identifies ultrasonographic site assessment as a major potential tool in predicting impending PIVC failure. We found that the presence of subcutaneous edema on ultrasound was a significant predictor of PIVC failure despite a normal clinical exam. Therefore, we were able to recognize that clinical exam was notably inadequate in identifying subcutaneous edema as only 6 patients experienced infiltration based on clinical assessment while 35 (56.45%) patients developed subcutaneous edema on ultrasound. Recent exploratory literature has suggested that subcutaneous edema represents more than a compromised or leaking vein due to infiltration. Instead, subcutaneous edema may represent a local inflammatory reaction due to ongoing mechanical and chemical insults to the vein wall [10,13,14]. Strategies targeting a reduction of subcutaneous edema may improve PIVC survival.

While other sonographic variables were not associated with a higher risk of PIVC failure, we noted some interesting associations that could be considered in future investigations. Prior research has shown that the location of the PIVC tip may be a potential factor in failure. While prior work has regarded this as a static variable, our methodology of serial assessments helped us discover that catheter tip location varies over the device’s lifespan. In 44 (71%) cases, the catheter tip to wall distance varied between daily evaluations. In 87% of cases, the catheter tip contacted the vein wall at least once during its lifespan, illustrating that vein irritation from PIVC tip likely occurs in more catheters than previously reported [10]. Within our small cohort average distance of the PIVC tip to the vessel wall approached statistical significance as a predictor of PIVC failure. Among failed catheters, the average PIVC tip to vein wall distance was 0.3mm vs. 0.4mm in the survival group (P = 0.0561). Recent trials have implicated that mechanical irritation of the vein wall from the catheter tip is a strong predictor of ongoing venous inflammation. In an analysis on the location of catheter tip position within the vein, Murayama et al. found that contact of the tip against the vein wall was associated with subcutaneous edema on ultrasound [10]. Another study in an animal model found that modifying the catheter within the vein to reduce contact against the vein wall led to a 40% reduction in subcutaneous edema [14]. Our data and these findings suggest that modification of the PIVC tip position is likely another key component in reducing early catheter failure. Additionally, our observation that PIVC tip position is dynamic rather than static is a novel finding that must be considered when attempting to create solutions to improve PIVC survival. While prior interventions aimed at modifying PIVC tip position have focused on insertion technique, our research suggests that modification of the device itself may be more impactful [15].

Importantly, our results demonstrated that the presence of subcutaneous edema on ultrasound occurred significantly earlier than any external signs of PIVC failure. Our approach of utilizing daily ultrasonographic site assessments allowed us to track the onset and progression of these sonographic changes and compare them to standard methods of PIVC site assessment. Thus, we recognized that ultrasound findings indicative of impending failure were present nearly 30 hours earlier than our current method of PIVC site assessment. Early recognition of impending PIVC failure has enormous tangible benefits, particularly with respect to reducing treatment delays and decreasing hospital length of stay. One study noted that PIVC related complications led to a significantly increased hospital length of stay (5.9 days vs. 3.9 days) compared to patients without PIVC complications [16]. Early identification of an impending failure before it is clinically apparent allows the treatment team to take a proactive and organized approach and plan for ongoing vascular access needs, potentially avoiding an interruption or delay in therapy.

A better understanding of the ultrasonographic subcutaneous changes that take place prior to PIVC failure may also hold the key to improving catheter survival. As we gain further understanding of the progression of a PIVC towards failure, we may discover catheter or treatment modifications that reverse catheter failure. Further inquiry is needed to test interventions that may allow for PIVC salvage after recognizing impending failure.

This study had some limitations. The study had a small sample size and the effects of certain variables on PIVC failure may be underestimated, particularly factors with marginal significance. Further, the findings may not be generalized to all settings as the study was conducted at a large academic tertiary care center with a unique population. Additionally, if the PIVC failed or patients were discharged during off-hours, the final determination of whether a PIVC was considered a success or failure was based on documentation within the study participant’s medical record. In some instances, the researchers found PIVCs with sonographic signs of severe inflammation, but clinical staff was not available to perform bedside functionality assessment of the catheter. It is possible that there may have been a delay in the recognition of PIVC complications and failure in some cases. A number of systemic or PIVC related factors could impact the formation of subcutaneous edema. While we accounted for several of these variables, we likely did not include all relevant confounders. Further, we recognize that daily ultrasonographic assessment of a PIVC site has its challenges from a clinical standpoint. The current reproducibility of this type of assessment outside of a research environment is limited by cost, availability of equipment, and expertise of personnel. However, ultrasonographic subcutaneous assessment is a key step in improving our understanding of PIVC failure and therefore a necessary component within a research protocol. With additional investigations and innovation, this strategy of ultrasound assessments to predict PIVC failure could be broadly applied to clinical medicine in the future.

## Conclusions

In this single site prospective observational investigation, we identified that the ultrasonographic finding of subcutaneous edema has a strong correlation with PIVC failure. We also found that ultrasonographic findings are present much earlier than any external site changes, suggesting that subcutaneous evaluation using ultrasound of PIVCs may be useful in planning ongoing and future vascular access needs. Further research is needed to test strategies that may salvage a catheter once subcutaneous edema is evident on ultrasound.

## Supporting information

S1 Appendix Scan area of ultrasound probe. (TIFF)

S2 Appendix Ultrasound measurements. (TIFF)

S3 Appendix Short and long axis ultrasound images depicting changes within the vein and surrounding soft tissue. (TIFF)
