# Supplementary figures and images for: Early recognition of peripheral intravenous catheter failure using serial ultrasonographic assessments
Source: PLoS One. 2021 Jun 16;16(6):e0253243. doi: 10.1371/journal.pone.0253243 (PMC8208550; doi:10.1371/journal.pone.0253243)

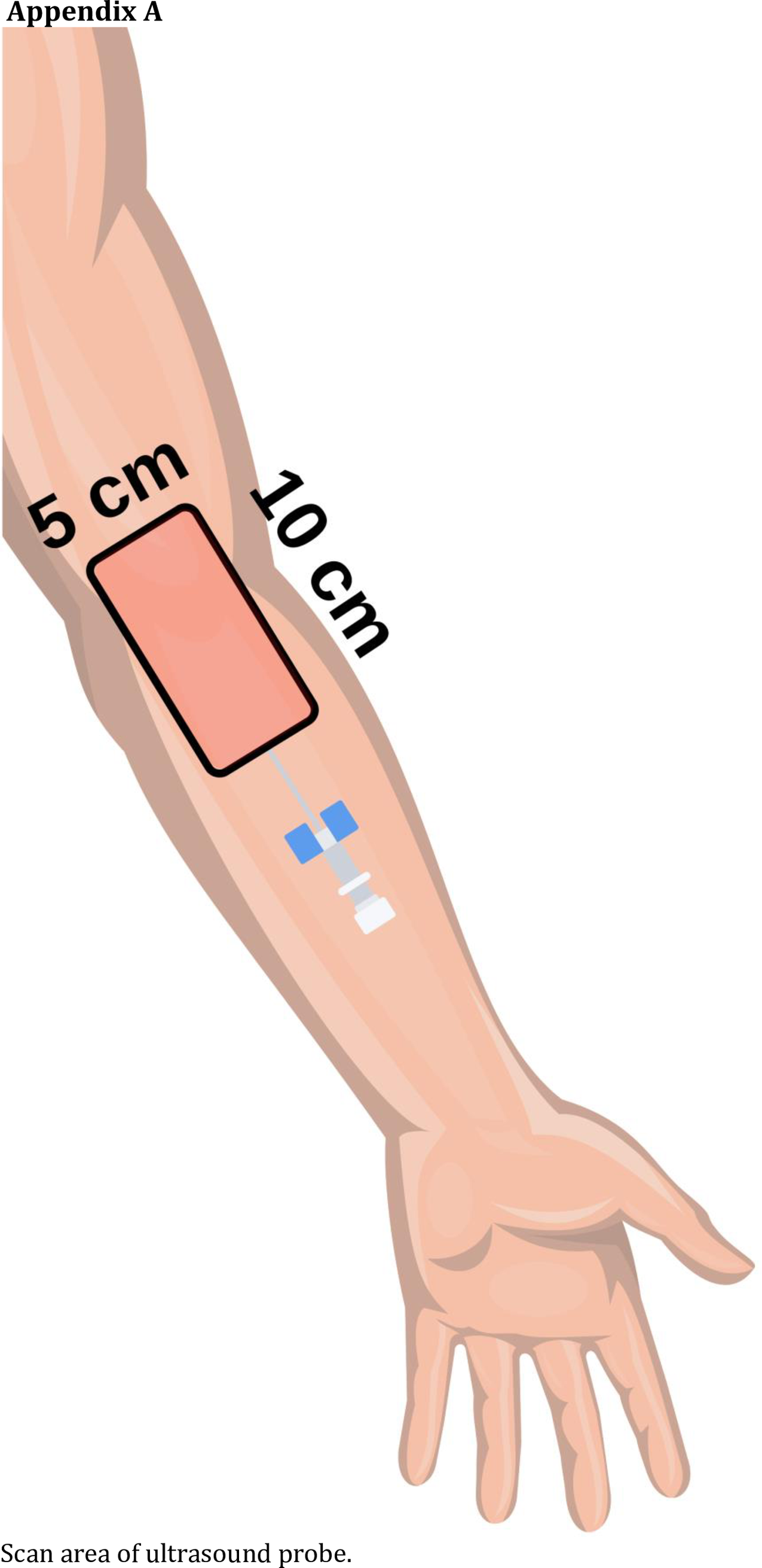

Supplement: S1 Appendix — (TIFF) [file pone.0253243.s001.tiff]

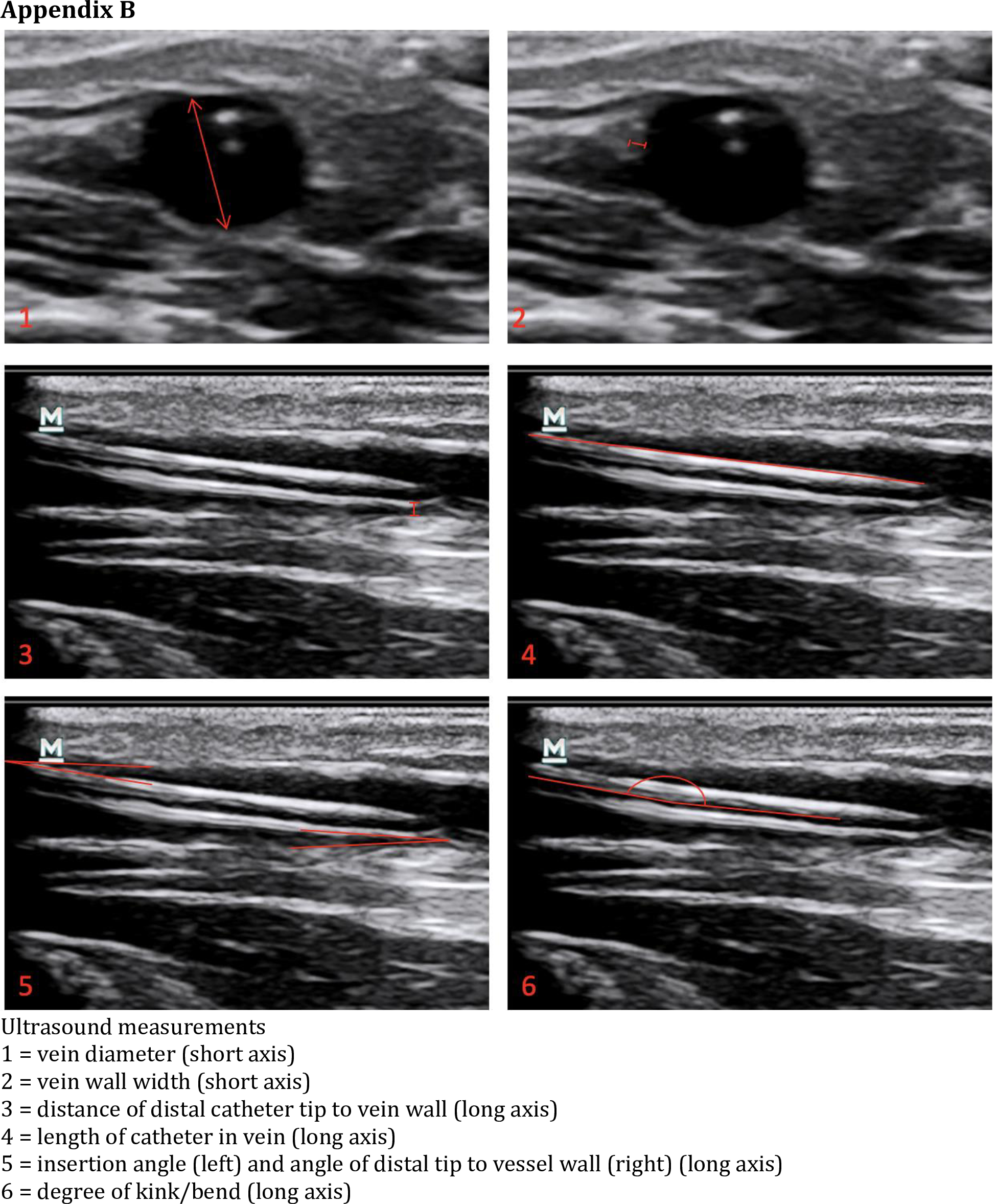

Supplement: S2 Appendix — (TIFF) [file pone.0253243.s002.tiff]

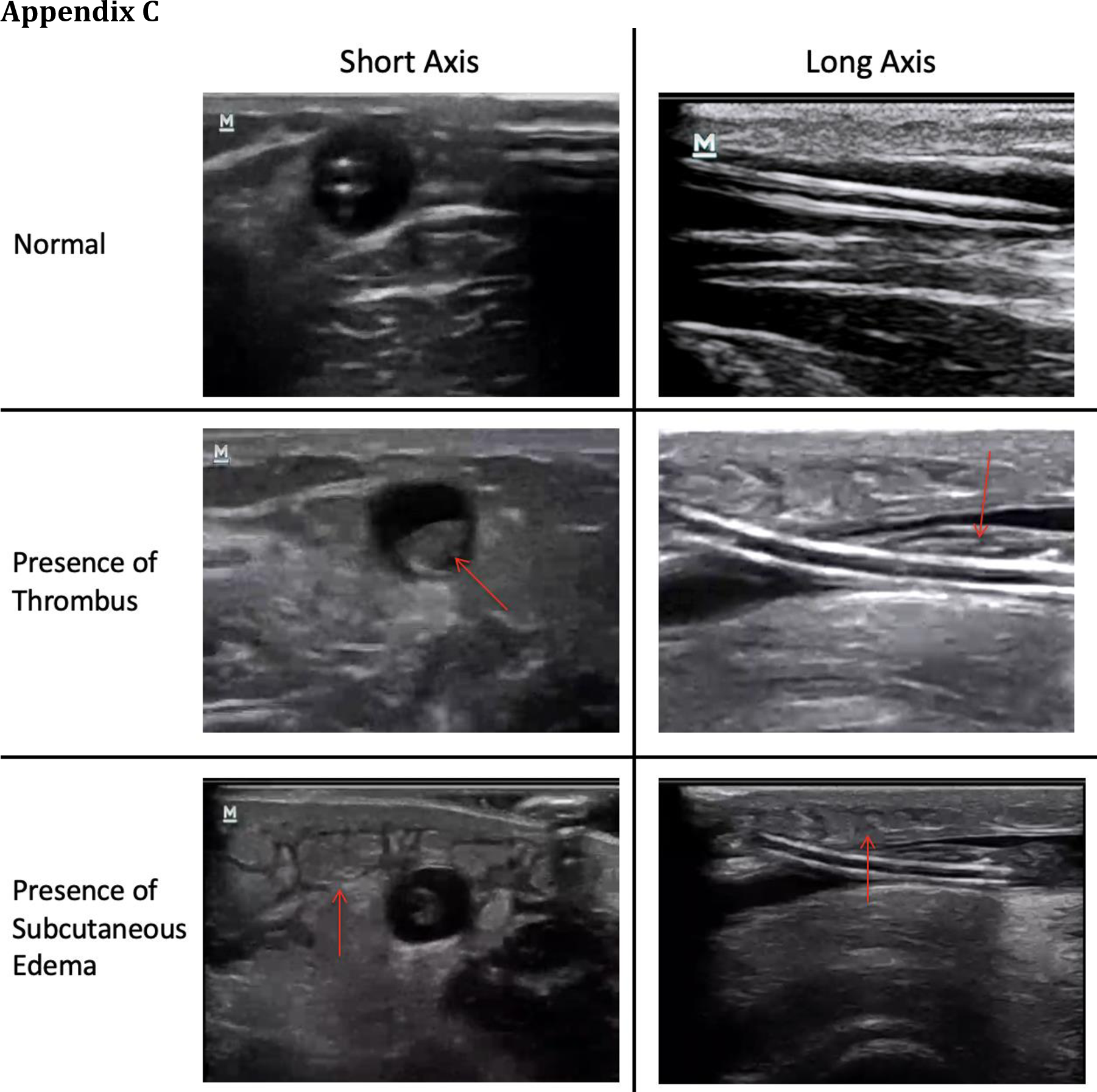

Supplement: S3 Appendix — (TIFF) [file pone.0253243.s003.tiff]
